# Fei-Liu-Ping ointment inhibits lung cancer growth and invasion by suppressing tumor inflammatory microenvironment

**DOI:** 10.1186/1472-6882-14-153

**Published:** 2014-05-12

**Authors:** Weidong Li, Cihui Chen, Shakir M Saud, Liang Geng, Ge Zhang, Rui Liu, Baojin Hua

**Affiliations:** 1 Oncology Department, Guang’anmen Hospital, China Academy of Chinese Medical Sciences, Beijing 100053, China; 2 Oncology Department, Zhejiang Provincial Hospital of TCM, Hangzhou, Zhejiang 310006, China; 3 Nutritional Science Research Group, Division of Cancer Prevention, National Cancer Institute, National Institutes of Health, Rockville, MD 21702, USA; 4 Henan Cancer Hospital affiliated to Zhengzhou University, Zhengzhou, Henan 450003, China

**Keywords:** Lung cancer, Fei-Liu-Ping ointment, Nuclear factor kappa light chain enhancer of activated B cells, Inflammation, Invasion, Epithelial mesenchymal transition, Cyclophosphamide

## Abstract

**Background:**

Lung cancer is one of the leading causes of cancer-related mortality worldwide. Conventional chemotherapy and radiotherapy are the primary therapeutic methods for lung cancer with the use of combination therapies gaining popularity. The frequency and duration of treatment, as well as, managing lung cancer by targeting multiple aspects of cancer biology is often limited by toxicity to the patient. There are many naturally occurring anticancer agents that have a high degree of efficacy and low toxicity, offering a viable and safe approach for the treatment of lung cancer. The herbs traditionally used in Chinese medicine for anticancer treatment offer great potential to enhance the efficacy of conventional therapy. In this study, we evaluated the synergistic effects of Fei-Liu-Ping (FLP) ointment in treating lung cancer; a known anticancer Chinese herbal based formula.

**Methods:**

In this study, A549 human lung carcinoma cell line and Lewis lung carcinoma xenograft mouse model were used. In addition, we utilized an *in vitro* co-culture system to simulate the tumor microenvironment in order to evaluate the molecular mechanisms of FLP treatment.

**Results:**

FLP treatment significantly inhibited tumor growth in the Lewis lung xenograft by 40 percent, compared to that of cyclophosphamide (CTX) of 62.02 percent. Moreover, combining FLP and CTX inhibited tumor growth by 83.23 percent. Upon evaluation, we found that FLP treatment reduced the concentration of serum pro-inflammatory cytokines IL-6, TNF-α, and IL-1β. In addition, we also found an improvement in E-cadherin expression and inhibition of N-cadherin and MMP9. We found similar findings *in vitro* when we co-cultured A549 cells with macrophages. FLP treatment inhibited A549 cell growth, invasion and metastasis, in part, through the regulation of NF-κB and altering the expression of E-cadherin, N-cadherin, MMP2 and MMP9.

**Conclusions:**

FLP exerts anti-inflammatory properties in the tumor microenvironment, which may contribute to its anticancer effects. FLP treatment may be a promising therapy for inflammation associated lung cancer treatment alone, or in combination with conventional therapies and may prevent lung cancer metastasis.

## Background

Lung cancer is the most commonly diagnosed malignancy and the main cause of cancer-related deaths worldwide, with an estimated 15 percent 5-yr survival rate [1,2]. While many advances in chemotherapy and radiotherapy have been made, overall patient survival rate has remained unchanged in recent years. During the early stages of lung cancer, surgery is effective; however, the five-year survival rate is approximately 25 percent, which is attributed mostly to remission and metastasis [3]. Preventing reoccurrence and metastasis is an important step in improving the survival for lung cancer patients. Minimizing the severity of some side effects can improve the success of therapeutic methods and improve patient quality of life.

Traditional Chinese medicine has low toxicity accompanied with few side effects, and offers a promising therapy for lung cancer patients [4,5]. Previous clinical and epidemiological studies demonstrated the efficacy of Traditional Chinese Medicine in treating lung cancer patients [6]. Experimental studies show that traditional Chinese medicine has multiple roles and targets in the treatment of lung cancer patients [7,8]; however, the mechanisms have yet to be elucidated.

Fei-Liu-Ping (FLP) ointment is an oral prescription medication that is used to treat lung cancer patients in China. FLP ointment has been shown to possess many anticancer properties [9], including improving the ability to stimulate dendritic cells [10], inhibiting VEGF [10] and modulating MMP9 and TIMP-1 expression [9]. The effects of FLP treatment on the tumor microenvironment have yet to be investigated.

The tumor microenvironment is an area of immense research; unlike normal cells, the microenvironment of cancer cells is hypoxic, acidic, hypertensive, with a greater number of growth factors secretion and proteolysis enzymes that promote proliferation and cell survival [11]. In addition, the tumor microenvironment is in a chronic inflammatory state that has been shown to play a critical role in tumor growth, progression, and metastasis [12].

In the clinic, we have found that FLP ointment relieves the symptoms manifested in patients with lung cancer that may be associated with inflammation. We hypothesized that FLP ointment could alleviate inflammation within the microenvironment; thus, preventing progression and metastasis. In this study, we were able to show that FLP inhibits tumor growth in a Lewis lung cancer xenograft, and inhibit migration, invasion and metastasis in A549 lung cancer carcinoma cell lines under inflammatory conditions.

## Methods

### Materials

FLP ointment was supplied from Pharmaceutical preparation center of Guang’anmen Hospital, China Academy of Chinese Medical Sciences. (No.(98)JWY[058] DF-1657) (Beijing, China). Cyclophosphamide (CTX) was supplied by Shanxi Powerdone Pharmaceutics Co. (Shanxi, China). Antibodies: NF-κB (p65), E-cadherin, N-cadherin, and MMP9, were purchased from Abcam (Cambridge, MA, USA). β-actin was purchased from Cell Signaling Technology (Danvers, MA, USA). ELISA kits for IL6, IL1β, and TNF-α were purchased from R&D systems (Minneapolis, MN, USA).

### Preparation of FLP ointment

The herbs used in FLP ointment are the roots of *Astragalus membranaceus (Fisch.) Bge.var.mongholicus (Bge.) Hsiao* (Huang-Qi), *Panax quinquefolium L. *(Xi-Yang-Shen), *Ophiopogon japonicas (Thunb.) Ker-Gawl.* (Mai-Dong), *Glehnia littoralis Fr. Schmidt ex Miq.* (Bei-Sha-Shen), *Agrimonia pilosa Ledeb. *(Xian-He-Cao), *Polygonum bistorta L.* (Quan-Shen), *Patrinia villosa (Thunb.) Juss.* (Bai-Jiang-Cao), *Panax notoginseng (Burk.) F.H. Chen* (San-Qi), *Fritillaria cirrhosa D. Don* (Chuan-Bei-Mu), *Glycyrrhiza uralensis Fisch.* (Gan-Cao), *Cordycrps sinensis(Berk.) Sacc.* (Dong-Cong-Xia-Cao) and the fruits of *Prunus persica (L.) Batsch* (Tao-Ren) and *Prunus armeniaca L. var ansu Maxim.* (Xing-Ren). All those herbs were from Guang’anmen Hospital according to the original proportion, and decocted twice with 8-fold volume of distilled water for 1 hour. The decoction were collected, filtered, merged and concentrated to 2 g/mL (equivalent to crude herb materials), and stored at 4°C for oral using.

### Animal studies

All mouse experiments were agreed to and regulated by the Ethical Committee of China Academy of Chinese Medical Sciences. Pathogen free male C57BL/6 mice were purchased from Vital River Company (Beijing, China) at 6-weeks of age and were maintained in a temperature- and humidity-controlled animal facility with a 12-h light/dark cycle. Animals were given free access to water and are monitored closely for any clinical signs of poor health throughout the study. All the animal experiments were conducted in accordance with the guidelines of the National Institutes of Health for the care and use of laboratory animals. After one-week acclimation, mice were randomly sorted into 5 treatment groups of 10 animals: Non-tumor-bearing (NT), Vehicle control (PBS) Fei-Liu-Ping ointment treated (FLP), cyclophosphamide treated (CTX), and FLP + CTX treated. Xenografts were performed under anesthesia by injecting 2 × 10^6^ Lewis lung cancer cells in 0.1 ml volume into the right flank of the mouse. The following day, mice were treated according to group. FLP mice were treated orally with 100 mg/kg FLP by gavaging each day, for 21 days. CTX group mice were given three IP injections of CTX at 60 mg/kg, on day 2, 7, and 14 following tumor injection. FLP + CTX group mice were given a combination of CTX and FLP treatments described above. The vehicle control group received PBS gavaging each day, for 21 days. Mice were euthanized by CO_2_ asphyxiation on day 21 and tumor tissues were collected, measured and weighed. Serum from each mouse was also collected into 1.5 ml sterilized micro-tubes and kept on ice for 40 minutes, then centrifuged at 4°C at 3000 RPM for 15 minutes, and stored at -80°C for further analysis.

### Tumor inhibition rate

Tumor inhibition rate was calculated as follows: Tumor inhibition rate = (1- average weight of tumors in treatment group/average weight of tumors in control group) × 100%.

### Immunohistochemistry

Paraffin embedded tumor tissue was sectioned at 5 μm, onto glass microscope slides and subjected to immunohistochemical analysis. Briefly, tissue sections were deparaffinized in xylene and rehydrated in graded ethanol. Sections were then subjected to antigen retrieval and blocked for 1 hour in normal serum. Sections were incubated in primary antibody solution at the following dilutions, NF-κB, 1:50; N-cadherin, 1:100; E-cadherin, 1:200; MMP9, 1:200, overnight. This was followed by incubation in a horseradish peroxidase (HRP)-conjugated secondary antibody (Cell Signaling Technology) for 1 hour. Sections were visualized with 3, 3’diaminobenzidine (DAB) (Millipore, Billerica, MA, USA) and lightly counterstained with hematoxylin. Expression of positive cells was performed using image analysis software ImageProPlus 4.5 (Diagnostic Instruments, USA).

### Western blot analysis

Western blot analysis of protein isolated from tumor tissue was performed as follows. Briefly, 100 mg tumor tissue from each sample was homogenized in 1 ml radioimmunoprecipitation assay (RIPA) lysis buffer (Thermo scientific, Waltham, MA, USA). Protein concentration was calculated using standard bovine serum albumin (BSA) curve. Approximately 30 μg of protein was loaded and fractionated by SDS-PAGE and transferred to PVDF membrane and probed with the following primary antibodies at the indicated dilution. NF-κB (1:1000), E-cadherin (1:1000), N-cadherin (1:1000), MMP9 (1:500), and β-actin (1:5000) followed by a horseradish peroxidase (HRP)-conjugated secondary antibodies. Signal was visualized by adding ECL detection reagent (Amersham Life Science, Piscataway, NJ, USA).

### Cell culture

A549 human adenoma cell line was obtained from cell resource center of the Institute of Basic Medical Sciences Chinese Academy of Medical Sciences. Cells were cultured in RPMI1640 with 10% FBS, 100U/ml penicillin and 100 mg/ml streptomycin at 37°C in humidified air with 5% CO_2_. THP-1 human monocyte cell line was obtained from cell resource center of the Institute of Basic Medical Sciences Chinese Academy of Medical Sciences and cultured in RPMI1640 with 10% FBS at 37°C in humidified air with 5% CO_2_.

To study the effects of Fei-Liu-Ping (FLP) ointment *in vitro* that closely resembles the serum bioavailability of FLP treatment *in vivo,* we utilized serum pharmacology previously described in [13-16]. Briefly, Wistar rats, a strain of albino laboratory rats that are often used to study safety and efficacy testing, were subjected to saline, FLP, or CTX treatment. Animals received either oral gavage of saline treatment, or FLP treatment at a low, medium or high dose (50, 100, 200 mg/kg, respectively), or IP injections of CTX (30 mg/kg) twice a day for three consecutive days. On the third day, serum was collected from the animals, filtered through a 0.2 μm cellulose acetate membrane. The serum collected was used for the treatments in all downstream *in vitro* assays and listed as follows; NRS (normal rat serum, saline treated), FLPL (FLP low dose), FLPM (FLP medium dose), FLPH (FLP high dose).

### In-vitro system of inflammatory microenvironment

THP-1 cells were seeded on 6-well plates at 1 × 10^6^ cells/well. The THP-1 monocyte cells were converted to macrophages using Phorbol 12-Myristate 13 acetate (PMA) purchased from Sigma Aldrich (Shanghai, China) at a final concentration of 320nM and incubated at 37°C with 5% CO_2_ for 48 hours to allow for maximal conversion of the THP-1 monocytes to THP-1 macrophages. All the experiments were performed in polystyrene with Transwell inserts with a pore size of 0.4 μm from BD Biosciences (San Diego, CA USA) using serum free medium. The co-culture experiments consisted of adding an insert containing confluent THP-1 macrophages to cultured A549 cells that are grown in the bottom compartment of the plate. The THP-1 macrophages were never in direct contact with the A549 cells. The cultures were exposed to either vehicle control or various FLP treatments. Cultured A549 without the addition of THP-1 macrophages was used as an experimental control. After 24 hours, the medium was recovered and stored at -80°C for further analysis.

### ELISA detection of inflammatory cytokines

ELISA analysis was performed according to the manufacturers’ instructions and analyzed using a spectrometer reading at an absorbance of 450 nm with 570 nm used as the reference wavelength (Synogen4, Gene company Ltd).

### Cell proliferation assay

A549 cells were incubated in 96-well plates in the presence of FLP or CTX treatments for 48 hours. MTT (3-[4, 5-dimethylthiazol-2-yl]-2, 5 diphenyl tetrazolium bromide) purchased from Sigma-Aldrich (Shanghai, China) was added for 4 h at the final concentration of 0.5 mg/ml. Subsequently, the culture medium was removed and after dissolving the formazan crystals in DMSO, plates were read immediately at 570 nm using an absorbance plate reader (Bio- Rad, CA, USA). Cell survival was defined as the growth of treated cells compared with untreated cells.

### Scratch wound assay

A549 cells were grown to confluence on a 24-well dish in serum-free medium. A single stripe (500 μM wide) was scraped on the cell-coated surface with a disposable plastic pipette tip. Cells were treated with FLP or CTX and incubated for 24 hours at 37°C with 5% CO_2_. Migration was analyzed using light microscopy with Image-Pro Plus Version 6.0.

### Invasion assay

Cell invasion was determined using Matrigel invasion chambers purchased from BD Biosciences (San Diego, CA, USA) and according to the manufacturers’ instructions. Briefly, 1 × 10^4^ A549 cells were seeded into the upper chamber in serum free medium separated by a permeable membrane. The bottom chamber contained 10% serum in the presence or absence of FLP or CTX. Serum-free medium in the bottom chamber was used as a control. After 24 hours the cells in the membrane was fixed, stained and counted using light microscopy.

### Immunofluorescent staining

After pre-treatment with FLP or CTX, A549 cells were fixed for 20 minutes using 4% formaldehyde in PBS. The cells were blocked in 1% bovine serum albumin with 0.02% Triton X-100 for 1 hour and then incubated with primary antibodies, NF-κB (p65) (1:200), E-cadherin (1:200), N-cadherin (1:200), MMP9 (1:100), MMP2 (1:100), overnight at 4°C followed by a 1 hour incubation in Alex Fluor^594^ secondary antibody (1:400) and counterstained with DAPI. Molecular DevicesMetaXpress® Image Acquisition and Analysis Software were used for analysis of translocation of NF-κB p65, E-cadherin, N-cadherin, MMP2 and MMP9.

### Statistical analysis

All data is presented as the mean ± standard deviation. The significance of the difference between groups was evaluated by one-way repeated-measures analysis of variance (ANOVA) and multiple comparisons with Prism 5.0 software. Student’s *t*-test for paired observation was used where appropriate. *P* < 0.05 were considered to be statistically significant.

## Results

### FLP treatment inhibits tumor growth in Lewis lung cancer xenograft mice

**Figure 1:**
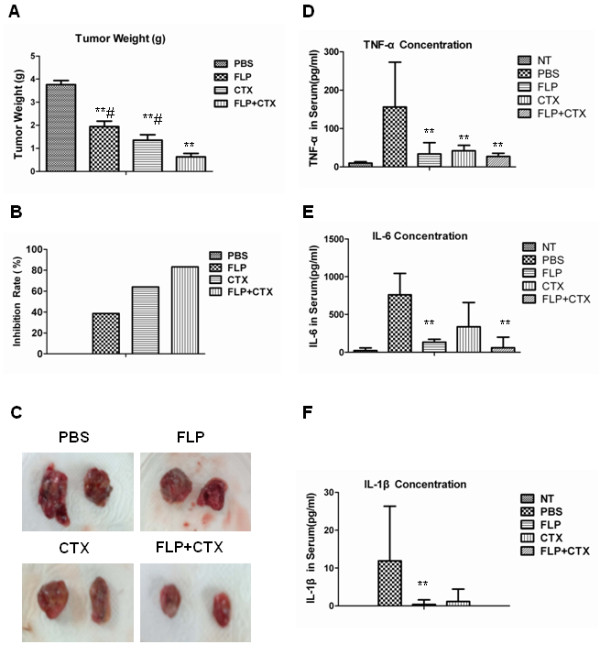
**Fei-Liu-Ping ointment inhibits the tumor growth.** FLP effects on tumor growth in Lewis lung cancer xenograft. Lewis lung cancer cells were injected into 6-week-old C57BL/6 mice (2 × 10^6^ cells/mouse). FLP mice were treated orally with 100 mg/kg FLP by gavaging each day, for 21 days. CTX group mice were given three IP injections of CTX at 60 mg/kg, on day 2, 7, and 14 following tumor injection. FLP + CTX group mice were given a combination of CTX and FLP treatments described above. **(A)** Tumors were weighed (grams) at the end of 21 days. Data displayed as mean of 10 mice in each group, +/- standard deviation. ^#^*P* < 0.05 compared to FLP + CTX; **, *P* < 0.01 compared to PBS control. **(B)** Tumor Inhibition rate calculated as percent compared to PBS control. **(C)** Representative photomicrographs of tumors. Serum cytokine analysis detected using ELISA plotted as concentration for **(D)** TNF-α **(E)** IL-6 **(F)**, IL-1β. Data displayed as mean of 10 separate samples in each group +/- Standard deviation. **, P < 0.01 compared to PBS vehicle.

To measure tumor growth inhibition caused by FLP ointment treatment Lewis lung cancer xenograft mice were treated with PBS, FLP ointment, CTX or a combination of FLP ointment and CTX. At the end of the study, the mice were euthanized and tumor tissues were measured and weighed. Figure 1A shows that FLP ointment inhibited the tumor growth compared with PBS vehicle group (*p < 0.01*). CTX could also significantly inhibit the tumor growth compared with PBS vehicle group (*p < 0.01*). FLP ointment combined with CTX was more effective in reducing the tumor growth compared with FLP ointment alone, or CTX alone. As shown in Figure 1B and C, FLP inhibition rate was 40 percent compared with PBS vehicle group; CTX inhibition rate was 64.02 percent compared with PBS vehicle group; the inhibition rate for FLP combined with CTX was 83.23 percent. From the above results, we found FLP ointment could inhibit the tumor growth of Lewis lung cancer, by itself or when combined with other conventional chemotherapeutic agent, CTX.

### FLP treatment inhibits tumor-induced inflammatory cytokines expression in serum of Lewis lung cancer xenograft mice

FLP ointment was first prescribed in the clinic to lung cancer patients to relieve symptoms associated with inflammation, such as, cough, phlegm, and blood in the phlegm. Deregulation of inflammatory cytokines has been previously found to be associated with the above symptoms [17-20]. Serum collected from euthanized mice was analyzed for pro-inflammatory cytokines using ELISA. We found minimal detection of pro-inflammatory cytokines in the serum of the NT group (normal mice without tumor), whereas, tumor bearing mice had significantly elevated levels of TNF-α, IL-6 and IL-1β (Figure 1D, E and F). Interestingly, treatment with FLP decreased these cytokines to similar levels seen in normal mice without tumors. While a similar decrease in TNF-α and IL-1β was seen in the CTX treated mice, FLP treatment decreased IL-6 levels to a greater degree compared to that of the CTX treatment. Our results suggest that inflammation is induced by the tumor in Lewis lung cancer xenograft mice and FLP treatment could prevent tumor-associated inflammation.

### FLP treatment reduces NF-κB expression in tumor tissue *in vivo*

**Figure 2:**
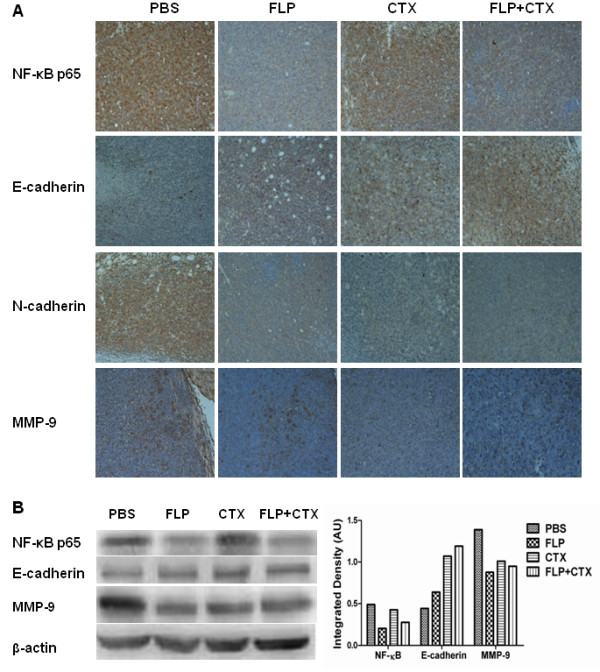
**Fei-Liu-Ping ointment decreases NF-κB expression, N-Cadherin, and MMP9 expression and increases expression of E-Cadherin *in vivo *.** Effect of FLP on the expression of NF-κB, E-cadherin, N-cadherin and MMP9. **(A)** Tumor tissue was collected from Lewis Lung xenograft was subjected to immunohistochemical analysis and stained for NF-κB, E-cadherin, N-cadherin, and MMP9 antibody, respectively. Data is shown as representative photomicrographs from each group (n = 10) at original magnification × 100. **(B)** Tumor tissue was collected from 10 mice per group, lysed and protein extracts were analyzed by Western blot for analysis for NF-κB, E-cadherin and MMP9. A representative blot is shown. Data analyzed using densitometry.

NF-κB is activated by inflammatory stimuli and is found to be constitutively active in several cancers, as a result, it has long been suspected to be a critical inflammatory promoter facilitating the initiation and progression of cancer [21]. NF-κB can induce pro-inflammatory cytokines, such as, IL-6 and TNF-α, and chemokine’s such as IL-8, adhesion molecules, MMPs, COX-2, and iNOS [22]. We analyzed tumor tissue collected from the Lewis lung xenograft by immunohistochemistry (Figure 2A) and western blot (Figure 2B) for NF-κB p65 expression. We found that FLP treatment can reduce the expression of NF-κB compared to the PBS vehicle group. Interestingly, CTX treatment was found not to have any effect on NF-κB expression; however, when CTX is combined with FLP we found a significant decrease in NF-κB expression.

### FLP treatment results in alterations of markers related to metastatic potential *in vivo*

Epithelial–Mesenchymal Transition (EMT) plays an important role in tumor invasion and metastasis [23]. The major characteristic of EMT is the transformation from epithelial type cells to more motile, invasive and migratory mesenchymal type cells [24]. In the process, epithelial cells lose cell-cell adhesion and cell polarity; In addition, decrease the expression of epithelial markers, such as, E-cadherin, and increase the expression of mesenchymal cell markers, such as, Vimentin, N-cadherin, alpha smooth muscle actin (α-SMA) [25]. In addition to EMT, deregulation of the extracellular matrix (ECM) and basement membrane by matrix metalloproteinase (MMPs), such as, MMP2, MMP9 is thought to play a key role in cancer cell invasion and metastasis [26,27]. The presence of macrophages and TNF-α secretion can also increase the expression of MMPs [28,29]. We analyzed the expression of E-cadherin, N-cadherin, and MMP9 in tumors collected from Lewis lung xenograft mice to measure the metastatic potential by immunohistochemistry (Figure 2A) and western blot (Figure 2B). As expected, we found altered expression of E-cadherin, N-cadherin, and MMP9 in PBS treated tumor bearing mice; Interestingly, FLP treatment, as well as, CTX treatment increased E-cadherin expression and decreased the expression of N-cadherin and MMP9, with a synergistic effect when FLP and CTX treatment was combined.

### FLP treatment inhibits cell proliferation *in vitro*

**Figure 3:**
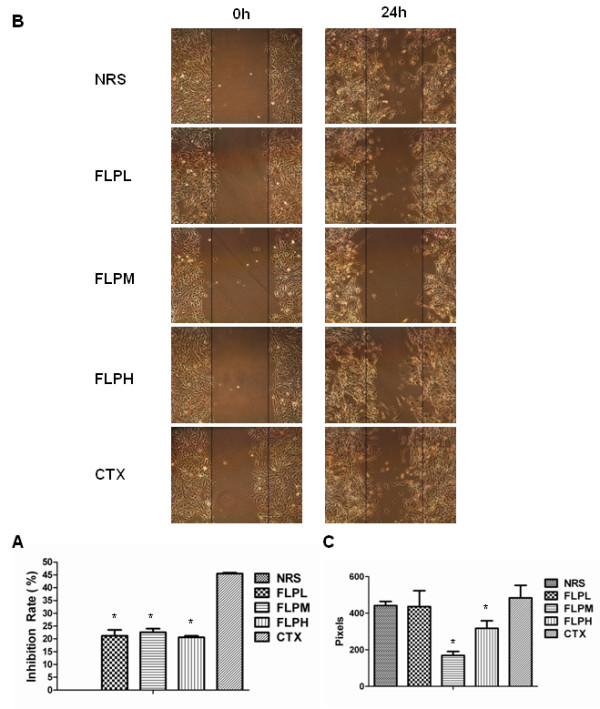
**FLP inhibits A549 cell proliferation and migration *in vitro.*** Effect of FLP on cellular proliferation and migration. **(A)** A549 cells were seeded in 96-well plates and treated with NRS (vehicle control), FLP treatment (low, medium, high) or CTX for 48 hours and subjected to MTT assay. Data are shown as mean ± standard error for three independent experiments. *P < 0.05 versus NRS. **(B)** A549 cells were co-cultured with THP-1 macrophages for 24 hours and treated with FLP at different dosages and subjected to scratch-wound healing assay. Representative photomicrographs of three independent experiments are shown and quantitated using imaging software measured as pixels **(C)**. *P < 0.05 versus NRS.

In order to further evaluate the molecular mechanisms of FLP anticancer activities, we treated A549 lung carcinoma cells, with FLP or CTX treated rat derived serum (see cell culture methods). We first evaluated the effects of FLP treatment on cell proliferation *in vitro*. Using a MTT cell proliferation assay we found that FLP treatment could inhibit cell proliferation in A549 cells with the low dose achieving significant inhibition; however, not to the same level of inhibition seen with CTX treatment (Figure 3A).

### FLP treatment inhibits cell invasion in an *in vitro* system of inflammatory microenvironment

**Figure 4:**
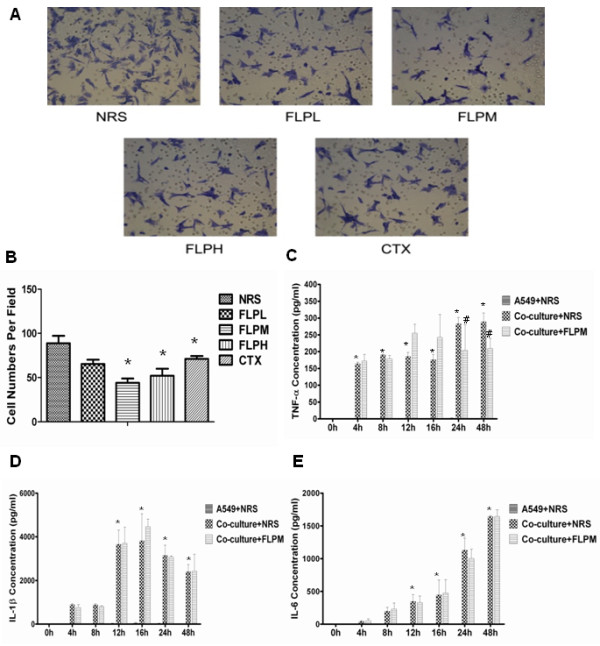
**FLP inhibits A549 cells invasion and inhibits pro-inflammatory cytokines when co-cultured with THP-1 macrophages.** Effect of FLP on A549 cell invasion and pro-inflammatory cytokines TNF-α, IL-1β and IL-6. **(A)** Representative photomicrographs of A549 cells in matrigel invasion assay treated with NRS (Vehicle control), FLP (low, medium high) or CTX, after 48 hours.** (B)** Image Pro Plus Version 6.0 software was used to calculate migrated cells for each treatment, 5 fields were randomly chosen to average migrated cells, all the experiments were repeated 3 times. *P < 0.05 vs. NRS group. Measurements for pro-inflammatory cytokines were measure by ELISA data displayed as concentration of **(C)** TNF-α, **(D)** IL-1β and **(E)** IL-6 in the supernatant. *P < 0.05 vs. A549 + NRS group.

Based on the earlier results from the xenograft studies, we found that inflammation may play a critical role in establishing the metastatic potential of lung tumors and that FLP may prevent migration and invasion; in part, by reducing pro-inflammatory cytokines and NF-κB. To directly test FLP efficacy in preventing invasion, possibly by modulating inflammation, we co-cultured A549 lung cancer cells with THP1-macrophages in a cell invasion assay. We found that a medium dose of FLP treatment could inhibit lung cancer invasion by 60% (Figure 4A, B). In addition, we found similar results in a scratch-wound migration assay (Figure 3B, C). We collected the supernatant from the co-culture medium at various times throughout the experiment to measure changes in pro-inflammatory cytokines. As expected, we found an increase in pro-inflammatory cytokines TNF-α, IL-6, and IL-1β when A549 cells are co-cultured with THP-1 macrophages. While, FLP treatment could reduce the level of TNF-α at 24 and 48 hours, the levels of IL-6 and IL-1β remained unchanged (Figure 4 C, D, and E). We concluded that FLP inhibition of invasion might be associated with changes in TNF-α.

### FLP treatment inhibits NF-κB activity and nuclear translocation *in vitro*

**Figure 5:**
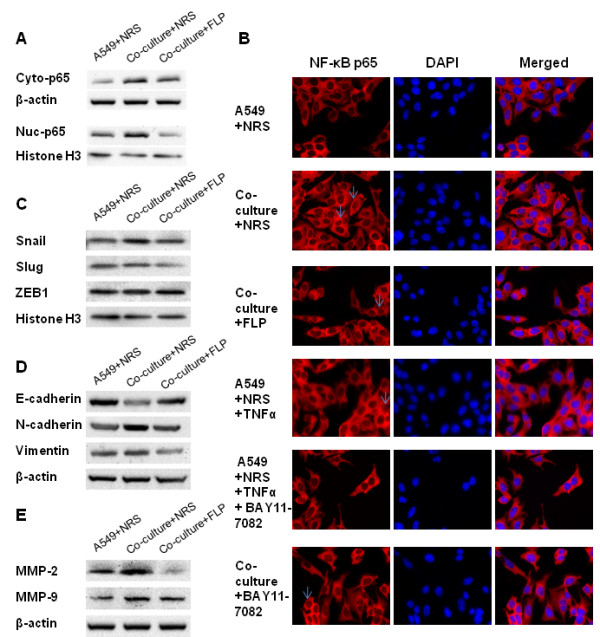
**FLP inhibits NF-κB translocation activity and EMT- associated protein *in vitro*.** Effect of FLP on NF-κB expression and EMT-associated proteins in A549 cells. **(A)** Western blot analysis of cytoplasmic and nuclear expression of NF-κB when A549 cells were cultured with or without macrophages, +/- FLP treatment, NRS (vehicle control). **(B)** Immunofluorescent staining of NF-κB localization following 24-hour of treatment counterstained with DAPI, representative pictures at original magnification × 400 were shown. **(C)** Western blot analysis of total protein for Snail, Slug, ZEB1 expression. **(D)** Western blot analysis of total protein E-cadherin, N-cadherin and Vimentin expression. **(E)** Western blot analysis of total protein MMP2 and MMP9 levels. All the experiments were repeated 3 times.

Based on the FLP inhibition of TNF-α concentration described above and the proposed role of TNF-α in activating NF-κB, we examined NF-κB expression in A549 cells using immunofluorescence. We found that we could induce the nuclear translocation of NF-κB by 16.9 or 13.34 percent by co-culturing A549 cells with THP-1 macrophages or with the addition TNF-α, respectively; Interestingly, we found a 11.15 percent decrease in the nuclear translocation of NF-κB with FLP treatment, compared to that of known NF-κB p65 inhibitor, BAY11-7082 with 5.93 percent inhibition (Figure 5A, B).

### FLP treatment reduces the expression of metastasis- associated proteins *in vitro*

**Figure 6:**
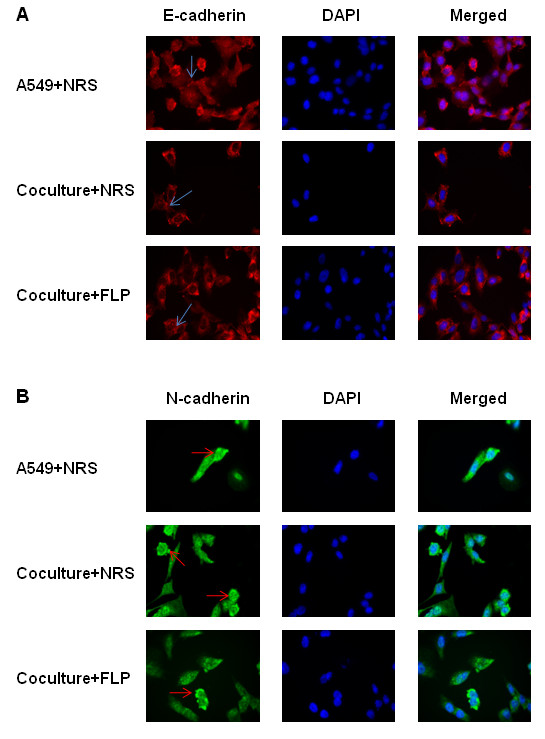
**FLP rescues E-cadherin and inhibits N-cadherin expression *in vitro*.** Effects of FLP treatment on E-cadherin and N-cadherin expression by immunofluorescence under inflammatory conditions. Photomicrographs of A549 cells stained with **(A)** E-cadherin, **(B)** N-cadherin, respectively counterstained with DAPI after pre-treatment with NRS (Vehicle control) or FLP. Representative pictures at original magnification × 400 were shown. All experiments were repeated 3 times.

**Figure 7:**
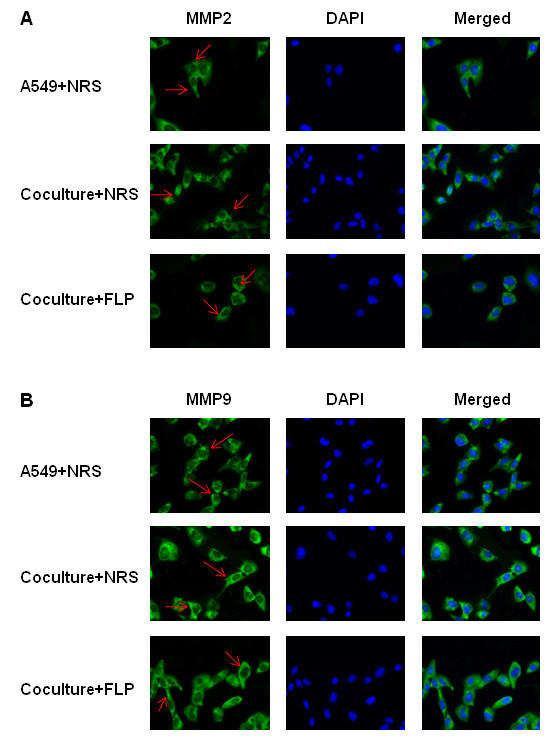
**FLP inhibits MMP2 and MMP9 translocation *in vitro*.** Effects of FLP treatment on MMP2 and MMP9 expression by immunofluorescence under inflammatory conditions. Photomicrographs of A549 cells stained with **(A)** MMP2 **(B)** MMP9, respectively counterstained with DAPI after pre-treatment with NRS (Vehicle control) or FLP. Representative pictures at original magnification × 400 were shown. All experiments were repeated 3 times.

NF-κB activity has been shown to regulate many downstream targets that may be associated with invasion and metastasis, including, transfer proteins, slug, snail, ZEB1, and E-cadherin, N-cadherin, and MMP9 as described above. Our xenograft study, implicated the influence of FLP treatment on several of the above markers, including, E-cadherin, N-cadherin and MMP9. We were able to support our *in vivo* findings by western blot showing that FLP treatment could increase the expression of E-cadherin and decrease the expression of N-cadherin and MMP9 (Figure 5D, E) in A549 cells cultured with THP-1 macrophages. In addition, we also found that FLP treatment could decrease the expression of snail, slug, ZEB1, Vimentin and MMP2, other proteins associated with metastasis (Figure 5C, D). We show similar results obtained by western blot with immunofluorescence, showing an increase in E-cadherin expression and decreased N-cadherin levels (Figure 6A, B), as well as, a decrease in MMP2 and MMP9 expression (Figure 7A, B).

## Discussion

Reducing tumor-associated inflammation and preventing reoccurrence and metastasis is an important therapeutic strategy for the management of lung cancer patients, and has posed a challenge for the medical community. Traditional Chinese Medicine offers great potential for enhancing current treatments for lung cancer, with minimum side effects. Although FLP ointment has been used to treat lung cancer and prevent lung cancer metastasis for many years in the clinic in China, the mechanisms are now being extensively investigated. In this study, we show that FLP treatment can inhibit tumor growth in a Lewis lung cancer xenograft study. FLP treatment inhibited tumor growth by approximately 40 percent; and when combined with chemotherapy drug CTX, inhibited tumor growth by 83.23 percent. Our results suggest that FLP treatment could improve the efficiency of chemotherapeutic drugs for the treatment of lung cancer.

Over the last decade, NF-κB has emerged as major pro-inflammatory mediator [30,31], and has been implicated in playing a role in carcinogen-induced lung inflammation, tumor formation, tumor invasion and metastasis [32-35]. As a result, many efforts are being made to target the NF-κB pathway. The predominant mechanism of NF-κB activation involves phosphorylation, ubiquitination, and degradation of inhibitors of NF-κB (IκB), allowing NF-κB dimers to translocate to the nucleus and promote transcription of target genes [36-38].

While the correlation between cancer invasion and NF-κB activity has been determined the molecular mechanisms is only poorly understood. It is thought that NF-κB promotes tumor progression, mainly by protecting transformed cells from apoptosis [39]. Some recent reports suggest that TNF-α is required for EMT and that NF-κB is essential for this process [40]. We demonstrate that FLP treatment could inhibit both TNF-α and NF-κB activity *in vivo* and *in vitro.* In A549 FLP treated cells, we found a greater inhibition of NF-κB than that seen with treating A549 cells with a known NF-κB p65 inhibitor, BAY11-7082. The inhibition of NF-κB by FLP was also associated with changes in expression of important mediators during EMT and invasion [40]. In both *in vivo* and *in vitro* models combining FLP with CTX rescued the altered expression of E-cadherin, N-cadherin and MMP9.

## Conclusions

Our results show that FLP ointment is effective in regulating inflammation within the tumor microenvironment, through interactions with the NF-κB signaling pathway and may inhibit lung cancer invasion and metastasis. FLP treatment combined with conventional therapies may possibly be used to target other NF-κB activated cancers, such as, glioblastoma. The current evidence warrant further investigation, nonetheless, offers promising potential to play an integral part in the treatment and management of lung cancer patients.

## Abbreviations

FLP: Fei-Liu-Ping ointment; NF-κB: Nuclear factor kappa light chain enhancer of activated B cells; EMT: Epithelial mesenchymal transition; CTX: Cyclophosphamide; MMP2: Matrix metalloproteinase 2; MMP9: Matrix metalloproteinase 9; TNF-α: Tumor necrosis factor alpha; IL-1β: Interleukin-1 beta; IL-6: Interleukin-6.

## Competing interests

The authors declare that they have no competing interests.

## Authors’ contributions

Conceived and designed the experiments: BH WL CC. Performed the experiments: WL CC LG GZ RL. Analyzed the data: WL CC SS. Contributed reagents/materials/analysis tools: WL CC LG. Wrote the paper: WL CC SS BH. All authors read and approved the final manuscript.

## Pre-publication history

The pre-publication history for this paper can be accessed here:

http://www.biomedcentral.com/1472-6882/14/153/prepub
